# Protective Effect of Folic Acid on Oxidative DNA Damage: A Randomized, Double-Blind, and Placebo Controlled Clinical Trial

**DOI:** 10.1097/MD.0000000000001872

**Published:** 2015-11-13

**Authors:** Xiaojuan Guo, Huan Cui, Haiyang Zhang, Xiaoju Guan, Zheng Zhang, Chaonan Jia, Jia Wu, Hui Yang, Wenting Qiu, Chuanwu Zhang, Zuopeng Yang, Zhu Chen, Guangyun Mao

**Affiliations:** From the School of Environmental Science & Public Health, Wenzhou Medical University, Wenzhou (XG, HZ, XG, CJ, HY, WQ, CZ, GM); School of Public Health, Inner Mongolia Medical University, Inner Mongolia (XG, ZZ); University Hospital of Wenzhou Medical University (HC); School of Laboratory Medicine & Life Science, Wenzhou Medical University, Wenzhou (JW); Center for Disease Control and Prevention of Wuyuan County, Inner Mongolia, China (ZY); Center on the Early Life Origins of Disease, the Johns Hopkins Bloomberg School of Public Health, Baltimore, MD (ZC, GM); and Center on Clinical & Epidemiological Eye Disease, the Affiliated Eye Hospital of Wenzhou Medical University, Wenzhou, China (GM).

## Abstract

Although previous reports have linked DNA damage with both transmissions across generations as well as our own survival, it is unknown how to reverse the lesion. Based on the data from a Randomized, Double-blind, Placebo Controlled Clinical Trial, this study aimed to assess the efficacy of folic acid supplementation (FAS) on DNA oxidative damage reversal.

In this randomized clinical trial (RCT), a total of 450 participants were enrolled and randomly assigned to 3 groups to receive folic acid (FA) 0.4 mg/day (low-FA), 0.8 mg/day (high-FA), or placebo (control) for 8 weeks. The urinary 8-hydroxy-2’-deoxyguanosine (8-OHdG) and creatinine (Cr) concentration at pre- and post-FAS were measured with modified enzyme-linked immunosorbent assay (ELISA) and high-performance liquid chromatography (HPLC), respectively. A multivariate general linear model was applied to assess the individual effects of FAS and the joint effects between FAS and hypercholesterolemia on oxidative DNA damage improvement. This clinical trial was registered with ClinicalTrials.gov, number NCT02235948.

Of the 438 subjects that received FA fortification or placebo, the median (first quartile, third quartile) of urinary 8-OHdG/Cr for placebo, low-FA, and high-FA groups were 58.19 (43.90, 82.26), 53.51 (38.97, 72.74), 54.73 (39.58, 76.63) ng/mg at baseline and 57.77 (44.35, 81.33), 51.73 (38.20, 71.30), and 50.65 (37.64, 76.17) ng/mg at the 56th day, respectively. A significant decrease of urinary 8-OHdG was observed after 56 days FA fortification (*P* < 0.001). Compared with the placebo, after adjusting for some potential confounding factors, including the baseline urinary 8-OHdG/Cr, the urinary 8-OHdG/Cr concentration significantly decreased after 56 days FAS [β (95% confidence interval) = −0.88 (−1.62, −0.14) and *P* = 0.020 for low-FA; and β (95% confidence interval) = −2.68 (−3.42, −1.94) and *P* < 0.001 for high-FA] in a dose-response fashion (*P*_trend_ < 0.001). Test of interaction between hypercholesterolemia and FA supplementation on urinary 8-OHdG reduction was significant (*P* = 0.001).

The present study demonstrates that FA fortification is independently linked to the reduction of urinary 8-OHdG/Cr in a dose-related pattern, which suggests that FA is beneficial to protect against oxidative damage to DNA. This effect is apparently stronger in those with hypercholesterolemia. The authors provide a new insight into the prevention and reversal of oxidative DNA damage.

## INTRODUCTION

Oxidative damage is a major contributor to many health problems, such as diabetes, atherosclerosis, and cancers.^1^ Almost all cellular components, including nucleic acids, are subject to oxidative damage. If left unrepaired, this damage can result in severely adverse cellular outcomes, including increasingly risk of mutagenesis and cell apoptosis.^2^ Compelling evidences support that oxidative damage is mainly induced by reactive oxygen species (ROS), such as hydrogen peroxide, superoxide anion, singlet oxygen, and hydroxyl radical, which can be produced through normal cellular metabolism or induced by a wide range of environmental factors.^3–6^

During the last several decades, it has been recognized that ROS can directly or indirectly damage cellular DNA and protein.^7^ The hydroxyl radical, a major component of ROS, is generally assumed to be the critical reactive species that directly attacks DNA. The interaction of the hydroxyl radical with the nucleases of the DNA strand, such as guanine, leads to the formation of 8-hydroxyguanine or 8-OHdG.^8^ As the predominant form of ROS-induced DNA oxidative lesions, mainly released in urine and very stable, urinary 8-OHdG determination and quantitative analysis has become a pivotal biomarker for measuring the effect of oxidative damage to DNA and is widely considered as an independent risk factor for many diseases including cancers.^9–11^

Oxidative DNA damage, if not been reversed in time, may result in mutations generated during replication, cell death or senescence, or altered transcription of genes important to cellular function.^2^ Thus, it is important to seek effective interventions to prevent ROS-mediated damages. Available evidence shows that some vitamins, cysteine, and methionine may have ameliorative efficacy on the reversal of oxidative stress.^12^ Among them, folic acid (FA) is an essential water-soluble vitamin and key cofactor in one-carbon metabolism that can regulate many different pathways such as cell growth, differentiation, DNA-repair, apoptosis, and carcinogenesis prevention.^13^ Although oxidative stress and the role of FA in preventing oxidative stress-mediated damage have been previously studied in animals,^14^ there is a knowledge gap regarding the association of FA with reduced oxidative damage to DNA in human beings and the mechanisms by which this might happen. In the present study, we use the data from a randomized clinical trial “Efficacy and Safety of Folic Acid Supplementation Lowering Arsenic in a Chronic, Low-level Exposed Arsenic Population: a Randomized, Double-blind, Placebo Controlled Clinical Trial (NCT02235948)” to assess the protective effect of orally administered FA for 56 days on oxidative DNA damage reversal.

## METHODS

### Study Participants and Setting

**FIGURE 1:**
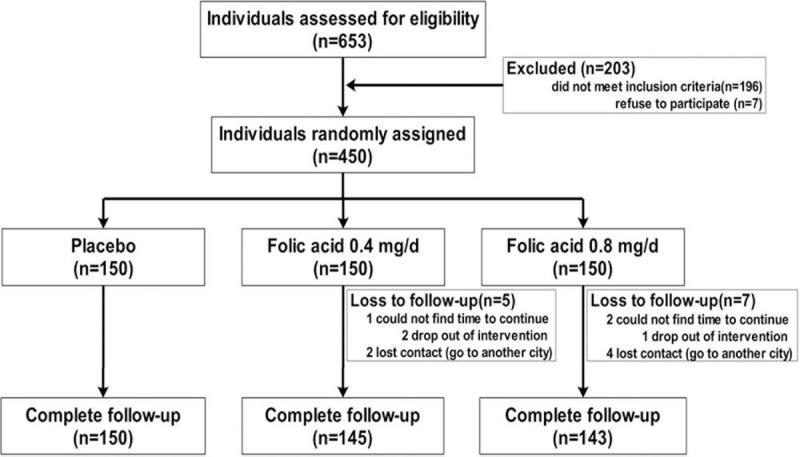
CONSORT flow diagram of the study participants.

The present study, was carried out between September 2010 and December 2011 in a population of 3 arsenic exposed villages stratified and randomly selected, based on the results of average arsenic concentration tests in the last 2 decades in Wuyuan county of Hetao Plain, Inner Mongolia, China (Fig. 1). Of 653 total residents in the above 3 villages, 450 (men 169; women 281) residents, at age of 18 to 79 years, were recruited for this folic acid supplementation (FAS) study, 203 were excluded because of not meeting the inclusion criteria (n = 196) or refusing to participate (n = 7). The inclusion criteria were: men or women more than 18 years of age and chronically exposed to arsenic (arsenic concentration of the drinking water >10 μg/L), those who had no FA supplementation in the 2 weeks before the study, women of childbearing age agreed to use a reliable contraception method during the study, and everyone volunteered to participate and signed informed consent. Patients were excluded if they were pregnant or breast-feeding women, were allergic to FA, had clearly defined allergic history, reported long-term use of FA and other B vitamins, had obvious signs, including gastritis, ulcer, etc. or laboratory abnormalities, which could affect the efficacy of FA or were otherwise deemed unsuitable to participate in the study based on the judgment of the investigators, did not agree to cancel the medications, which may affect serum folate concentration during the study period. In addition, subjects who were planning to become pregnant during the study or planned to move out of the area within the study period were also excluded. The protocol was approved by the ethics committee of Wenzhou Medical University, Wen-zhou, China. The purpose and procedure of the study were carefully explained to all participants. We got written informed consent from all of the participants before we began study-related procedures.

### Study Design

This study was a randomized, double-blind, placebo-controlled, parallel-group, clinical trial. Subjects and all study staffs were blinded to treatment group assignments.

### Randomization, Masking, and Intervention

After informed consent, we randomly assigned the eligible subjects in a 1:1:1 ratio according to the randomization schedule generated by statistician to one of 3 groups: 0.4 (low-FA), 0.8 (high-FA) mg FA per day, or placebo. To achieve a good balance among the 3 groups, permuted block randomization with a block size randomly selected as 6 was performed using R version 3.1.0 (Copyright 2011 the R Foundation for Statistical Computing). This statistician came from the School of Environmental Science & Public Health, Wenzhou Medical University, Wenzhou, China and was independent of study conduct and data analysis, and supplied to an independent GMP-certified Chang Zhou Pharmaceutical Company (Chang Zhou City, Jiangsu, China). Supplements were given as 2 pills to be orally taken once daily for 8 weeks and were provided free of charge. All pills, including different doses of FA or placebo were made indistinguishable by external color and appearance, labelled the identification number of each subject and distributed to the field study site by the pharmacy. The allocation of participants was encrypted, sent to the field study site, and accessible only to the staff in charge of the drug distribution. Participants and the study staffs were masked to the assignment all through the study duration. On the first day of the FAS period (visit 2), trained stuff allocated the pills to the subjects to eat according to the identification number. No request was ever made to break the blind during the study.

### Data Collection

All of the eligible subjects were followed for a total of 8 weeks. Face-to-face interviews were conducted at baseline, and in the fourth and eighth week of the study. The subjects were asked not to change their daily dietary habits and not to take any B vitamins or any other medications, which might affect the serum folate level during the following 8 weeks. All subjects underwent a physical examination at baseline and again at the end of the study (the 56th day of FAS). The physical examination was conducted by trained physicians from Wenzhou Medical University and Wuyuan CDC. All subjects were asked to fast overnight for 10 to 12 hours before the examination. A well-organized epidemiological survey was conducted by trained research staff according to the standard operating procedure on the first morning of the study. Each subject was interviewed with a standardized questionnaire designed specifically for the study, to collect demographic characteristic information on birth date, gender, occupation, education attainment, alcohol consumption, smoking habits, time spent in the area, history of drinking water intake, medical history, vitamin B usage, history of disease, and family history of diabetes, cardiovascular disease or dyslipidemia. Chronic arsenic exposure information was obtained from the subjects’ self-reporting, including type of drinking water and how long they had been drinking it. Height was measured without shoes to the nearest 0.1 cm, and weight was measured in light indoor clothing without shoes to the nearest 0.1 kg on portable weight scales. Body mass index (BMI) was calculated as weight (kilograms)/height (meters) squared. Seated blood pressure (BP) in all subjects was measured with the same type of mercury column sphygmomanometer between 8:00 and 10:00 a.m. All of the examinations, such as weight, height, seated BP, etc. were all measured 3 times by the same research staff at each time point and the mean values were used in the data analysis. Measures were taken to perform quality assurance and quality control during the study. All investigators and physicians had been trained for 2 weeks before the study commenced. All data collected from field studies, physical examinations, and laboratory assessments had to strictly follow the project standard operating procedure.

### Follow-up

During follow-up, treatment compliance evaluation (the percentage of participants following the study procedure) was performed in week 4 and 8. Good compliance was defined as taking 80% to 120% of the assigned supplements. “Loss to follow-up” was defined as the circumstance that occurs when researchers lose contact with the participants and cannot complete planned data collection efforts. Finally, we excluded 12 participants with missing values on any of the covariates and, thus, obtained a final study sample of 438 subjects (96.9%), and we analyzed all available data on baseline and 56 days later.

### Covariates

After 10 to 12 hours of fasting, 8 mL venous blood sample was obtained from each subject with tubes containing ethylenediaminetetraacetic acid for plasma, or tubes without ethylenediaminetetraacetic acid for serum at baseline and on the 56th day of the study. They were separated within 30 minutes and analyzed or stored at −86 °C in a freezer. Blood samples were collected for the assessment of serum lipids, including total cholesterol (TC), high-density lipoprotein (HDL) and triacylglycerols, creatinine (Cr), serum urea nitrogen, alanine aminotransferase, aspartate aminotransferase, plasma total homocysteine (t-Hcy) and serum FA concentration. Fasting plasma glucose was measured within 30 minutes. All of these tests were performed in the laboratory of Wuyuan CDC, using standard reagents and an automatic biochemistry analyzer. Low-density lipoprotein (LDL) was calculated by Friedewald's equation (LDL = TC − HDL − TG/2.2).

### Urine 8-hydroxy-2’-deoxyguanosine/creatinine Analyses

A total of 20 mL urine samples from each subject were also collected twice, at baseline (presupplementation) and on the morning of the 56th day (postsupplementation) at the same time point as the blood sample collection. These were put in a 0 °C ice box promptly, separated within 30 minutes, and stored at −86 °C in a freezer until they were sent to the central laboratory of the School of Laboratory Medicine & Life Sciences at Wenzhou Medical University packed in dry ice. For the measurement of urinary 8-OHdG concentrations, a modified enzyme-linked immunosorbent assay (ELISA)^15^ was used for the determination of 8-OHdG in urine by well-trained technicians in the central laboratory. To decrease the instability of the measurement, the urine 8-OHdG of each participant at baseline or the 56th day were assessed in triplicate. The mean value of 3 measurements was selected as the final 8-OHdG concentration. Urinary Cr was determined with high-performance liquid chromatography (HPLC) in duplicate by the same technician in the same lab and was applied to adjust for the bias induced by other factors in the urine. The level of 8-OHdG in urine was expressed as nanograms per milligram Cr and as the final outcome. The intra and interassay coefficients of variation were 3.4% and 4.1%, respectively.

### Statistical Analysis

We calculated the sample size of 132 participants per group with the assumption of a mean change of urinary 8-OHdG/Cr among the 3 groups for 0.00, 1.00, and 2.50 ng/mg after 8 weeks intervention with placebo or placebo, low-FA or high-FA and standard deviation (SD) 3.00 ng/mg, which provided 90% power to detect the differences with 1-way analysis of variance with a 2-sided type I error of 0.05. We added an additional 10% more participants to each group in view of possible lost during the follow-up. Finally, we recruited 450 subjects (150 per group) to conduct this study. This trial was registered with ClinicalTrials.gov, number NCT02235948.

**FIGURE 2:**
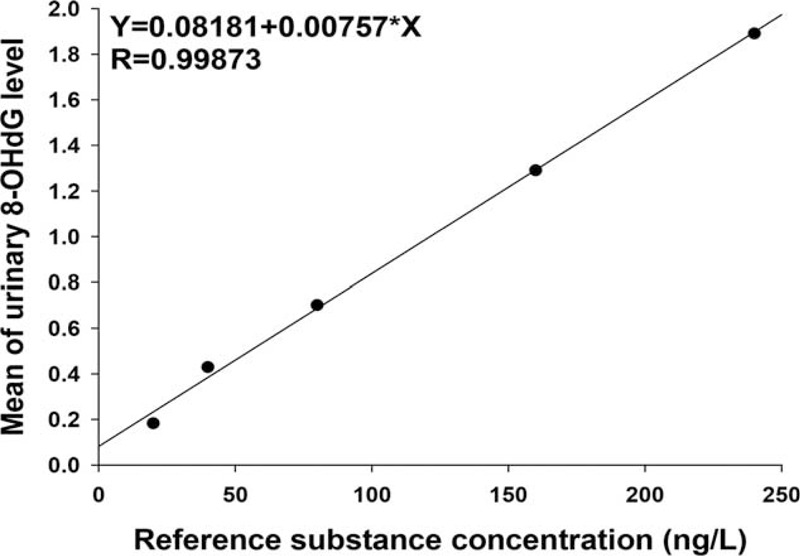
The standard curve for the urinary 8-hydroxy-2’-deoxyguanosine enzyme-linked immonosorbent assay.

Epidata (version 3.1, Denmark) was used to set up a dataset and conduct the double data entry, back to back, by the staff team at the School of Environmental Science & Public Health, Wenzhou Medical University. Data cleaning, such as veriﬁcation of the completeness and plausibility of data, was performed before unblinding, according to good clinical practice guidelines for clinical trials. The Shapiro-Wilk test was applied to assess the normality of data distribution before statistical analysis. Because urine 8-OHdG/Cr level at baseline and the end of the study were positively skewed, natural log-transformation was selected to increase the normalization of the distribution (Fig. 2). One-way analysis of variance for continuous variables and a χ^2^ test (or Fisher exact test) for categorical variables were applied to compare the characteristics of the study subjects in these 3 groups. Generalized linear mixed models were performed to compare the changes of urinary 8-OHdG/Cr in response to FAS, with the adjustment for corresponding baseline levels, age, sex, BMI, duration of arsenic exposure, and dietary habits, etc. All tests were 2-sided and *P* ≤ 0.05 was set as the signiﬁcant level. Data management and all statistical analyses were performed using R version 3.10 (Copyright 2014 the R Foundation for Statistical Computing) and figures were drawn by Sigmaplot 12.5 for Windows (SYSTAT Software Inc., Richmond, CA).

## RESULTS

### Characteristics of the Study Subjects

Between September 2010 and December 2011, 450 participants were recruited and randomized into 3 groups to receive different doses of FA or placebo (Fig. 1). The mean (±SD) age was 49.71 ± 12.36 years, and men constituted 37.56% (169/450) of the sample. Approximately 90% of the subjects included in the study were farmers and the duration of exposure to arsenic varied from 2.6 to 45.4 years (time of drinking the source water). More than half of them had no history of smoking or alcohol consumption and 80% of the subjects normally cooked using animal oil, which is a quite common practice in farming families in Inner Mongolia, and people with high blood cholesterol levels were also identified. A total of 438 of 450 participants completed the follow-up, 12 (low-FA: 5, high-FA: 7; placebo: 0) were lost because of not strictly following the protocol (n = 4) or were lost to follow up (n = 8). Baseline demographic, clinical, and biological characteristics were similar among 3 groups (Table 1). Moreover, the anthropometric and biochemical variables, such as BMI, BP, fasting plasma glucose, plasma lipid levels (TC), renal function, Cr, and arsenic-induced skin lesions showed no differences across these groups (all *P* > 0.05). Furthermore, we recorded 11 adverse events [5(3.33%) in the placebo, 2(1.37%) in the 0.4 mg/day, and 4(2.82%) in the 0.8 mg/day group] during the study. There was no significant difference among 3 groups (*P* = 0.537).

**TABLE 1:**
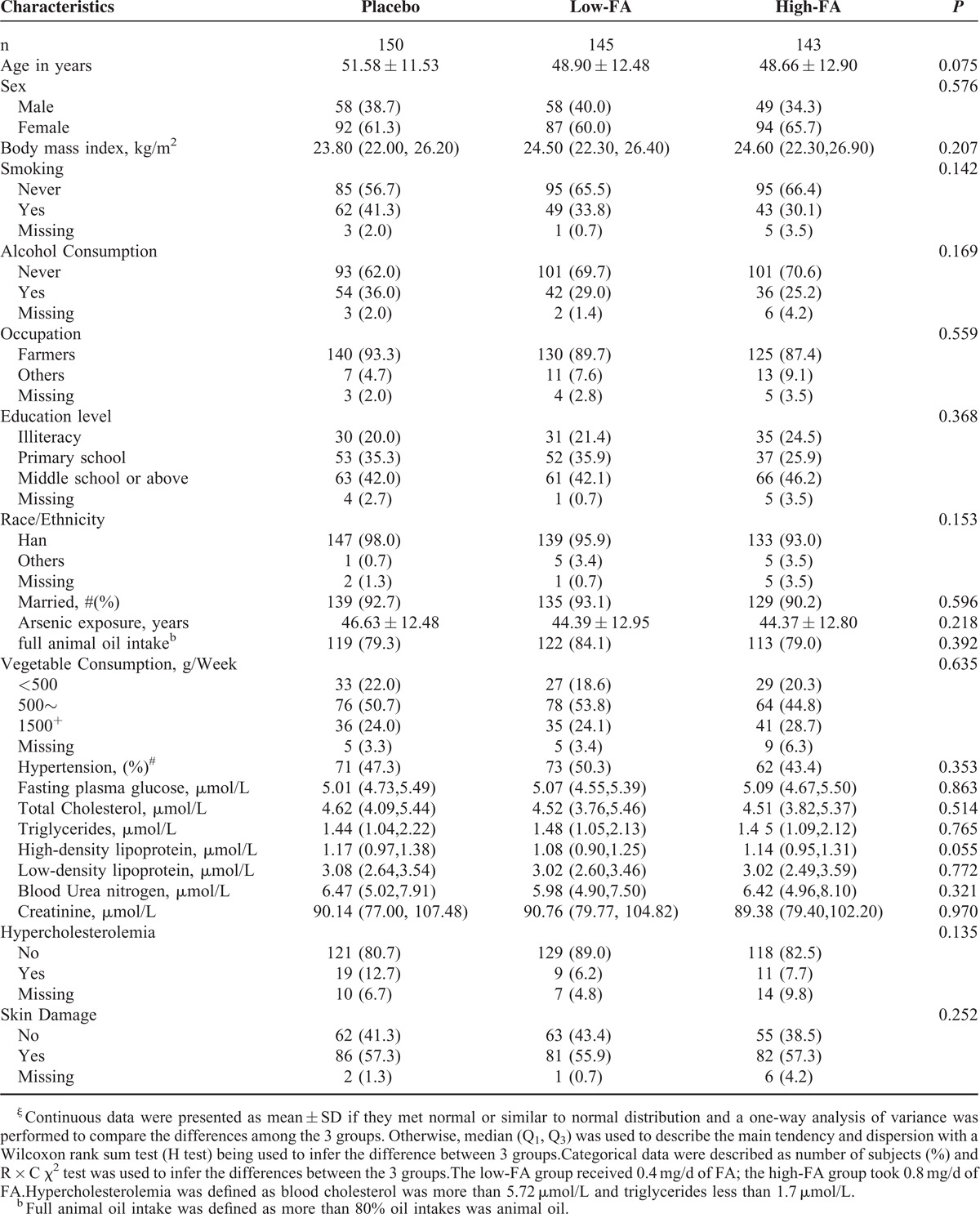
Characteristics of Study Subjects^ξ^

### Urinary 8-hydroxy-2’-deoxyguanosine/Creatinine Level in 3 Groups

**FIGURE 3:**
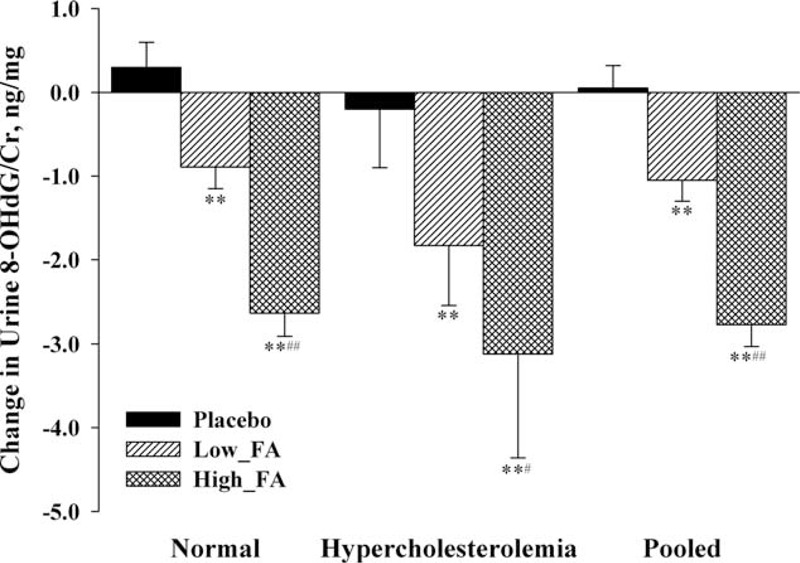
Comparison of 8-hydroxy-2’-deoxyguanosine/creatinine reduction among the 3 groups stratified by blood cholesterol level. Compared with placebo, ^∗^*P* < 0.05, ^∗∗^*P* < 0.01; Compared with Low-FA, ^#^*P* < 0.05, ^##^*P* < 0.01. 8-OHdG/Cr, 8-hydroxy-2’-deoxyguanosine/creatinine; Low-FA, 0.4 mg/d folic acid for 8 weeks; High-FA, 0.8 mg/d folic acid for 8 weeks. Hypercholesterolemia indicates that total blood cholesterol was more than 5.72 μmol/L and triglycerides less than 1.7 μmol/L; normal indicates that total blood cholesterol was less than 5.72 μmol/L and triglycerides more than 1.7 μmol/L.

The median and geometric mean value of urinary 8-OHdG levels of the study population before and after FA treatment are shown in Table 2. The concentration of urinary 8-OHdG/Cr at baseline was not significantly different among the 3 groups: the median (first quartile, third quartile) were 58.19 (43.90, 82.26) ng/mg in the placebo group, 53.51 (38.97, 72.74) ng/mg in the low-FA group, and 54.73 (39.58, 76.63) ng/mg in the high-FA group, respectively (*P* = 0.190). After taking placebo or FA for 56 days, the urinary 8-OHdG/Cr of the above the 3 groups was 57.77 (44.35, 81.33) ng/mg, 51.73 (38.20, 71.30) ng/mg, and 50.65 (37.64, 76.17) ng/mg, respectively. There was a significant decrease in the urinary 8-OHdG/Cr for those orally fortified with different dose of FA (Table 2 and Fig. 3). Meanwhile, we also computed the geometric mean and the antilog SD of the urinary 8-OHdG/Cr of the 3 groups at baseline and the 56th day because the data met lognormal distribution (Fig. 2). The geometric mean (±antilog SD) at baseline for the above the 3 groups was 60.82 ± 1.63 ng/mg, 55.48 ± 1.74 ng/mg, and 55.81 ± 1.72 ng/mg, respectively (*P* = 0.237). Although at the 56th day, the geometric mean (±antilog SD) were 60.82 ± 1.63 ng/mg for the placebo group, 54.27 ± 1.77 ng/mg for the low-FA group, and 52.40 ± 1.78 ng/mg for the high-FA group, respectively (*P* = 0.049).

**TABLE 2:**
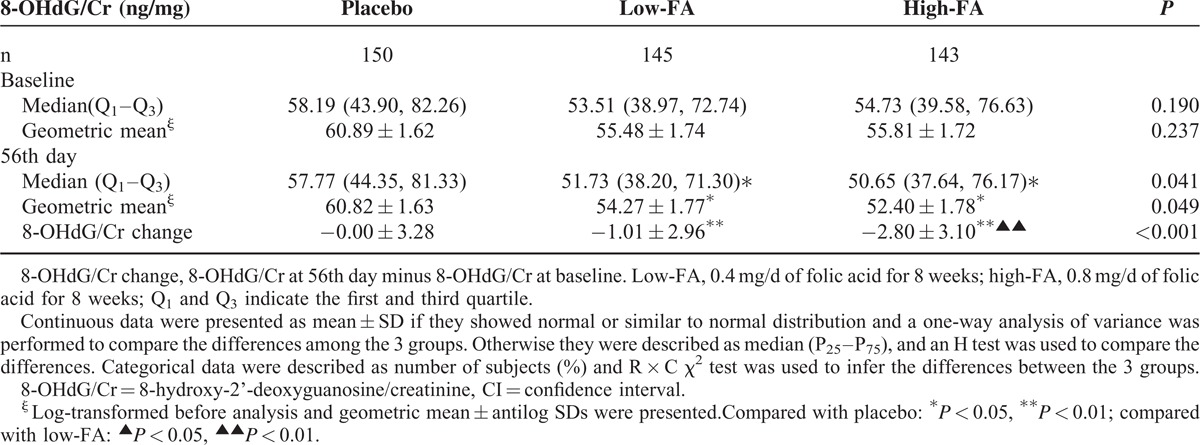
Comparison of 8-OHdG/Cr and the Change During Folic Acid Supplementation Among 3 Groups

### The Change in Urinary 8-hydroxy-2’-deoxyguanosine/Creatinine Levels with Folic Acid Supplementation

The change of the urinary 8-OHdG/Cr during the study was defined as the 8-OHdG/Cr concentration on the 56th day minus that at the baseline. There existed a significant difference in the change of 8-OHdG/Cr among the placebo, low-FA, and high-FA groups, which was 0.00 ± 3.28 ng/mg, −1.01 ± 2.96 ng/mg, and −2.80 ± 3.10 ng/mg, respectively (Table 2, *P* < 0.001). A multivariate generalized linear regression model was performed to assess the relationship between the change of urinary 8-OHdG/Cr and oral FA administration. After adjusting for all of the potential confounding factors including the baseline urinary 8-OHdG/Cr, supplementation with either 0.4 or 0.8 mg/day FA produced a significant decrease in 8-OHdG levels (*P* = 0.020 for 0.4 mg and *P* < 0.001 for 0.8 mg group) when compared with the placebo group (Table 3). We also found a clear dose-response relationship between the dose of FA and the decrease of urinary 8-OHdG/Cr based on another multivariate generalized linear regression model (trend test *P* < 0.001). Furthermore, the average reduction of urinary 8-OHdG/Cr in subjects with hypercholesterolemia fortified with FA was 0.96 ng/mg more than the change for those with normal blood cholesterol. Although the difference in the reduction of urinary 8-OHdG/Cr between subjects with or without hypercholesterolemia or not did not reach statistically significant levels (*P* = 0.175), perhaps because of the small sample size lacking power to identify the difference between the 2 groups, our results still indicate that blood cholesterol level has an impact on the efficacy of FAS on the change of 8-OHdG (Table 3).

**TABLE 3:**
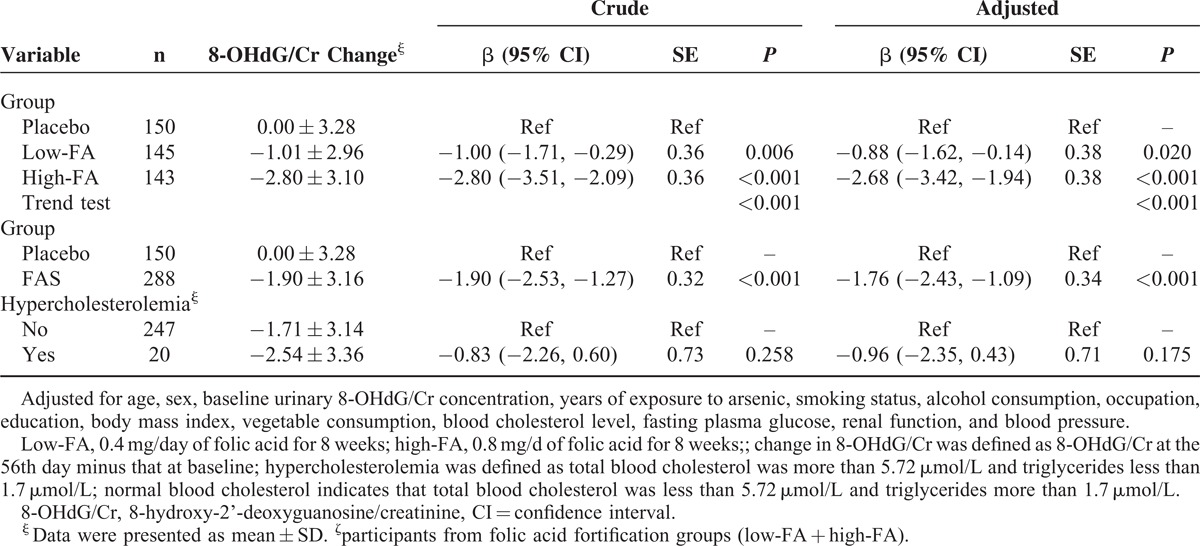
Individual Effect of Folic Acid Fortification or Blood Cholesterol Level on the Reduction of Urinary 8-Hydroxy-2’-Deoxyguanosine/Creatinine

### Joint Effect Between Folic Acid and Blood Cholesterol Level on the Reduction of Urinary 8-hydroxy-2’-deoxyguanosine/Creatinine

To robustly investigate the impact of blood cholesterol level on the efficacy of FAS in urinary 8-OHdG/Cr reduction, subanalysis was conducted stratified by subjects’ blood cholesterol level. As we can see from the Figure 3, subjects with either normal blood cholesterol level or hypercholesterolemia both showed significant reduction of 8-OHdG/Cr after FAS. A dose-response relationship between FA intake and urinary 8-OHdG/Cr was presented in both blood cholesterol levels of subjects. These results also seem to indicate that the efficacy of FAS on DNA oxidative damage reversal in people with higher blood cholesterol levels is even more pronounced.

**FIGURE 4:**
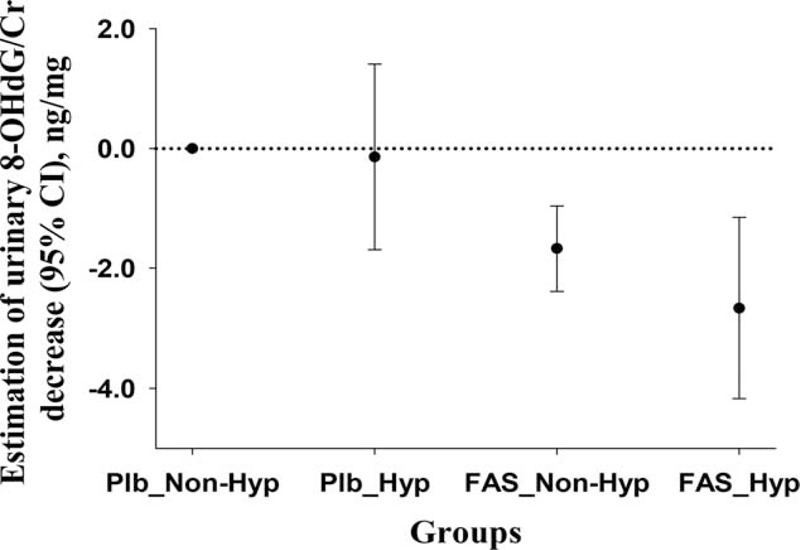
The estimated joint effects of folic acid supplementation and hypercholesterolemia on the reduction of urinary 8-hydroxy-2’-deoxyguanosine/creatinine. Plb, placebo; Nonhyp, nonhypercholesterolemia; FAS, folic acid supplementation; Hyp, hypercholesterolemia.

Furthermore, compared with the reference group (the subjects with no FAS and with normal blood cholesterol levels, see Table 4), the reduction in urinary 8-OHdG/Cr levels was 0.14 ± 0.79, 1.67 ± 0.36, and 2.66 ± 0.77 ng/mg in subjects who did not take FA but had hypercholesterolemia, and subjects who took FA but had normal cholesterol levels, as well as subjects with hypercholesterolemia and FAS, respectively. It is of interest to note that FA fortification and blood cholesterol levels jointly affect the reduction of urinary 8-OHdG/Cr (*P*_interaction_ = 0.001; Table 4 and Fig. 4).

**TABLE 4:**
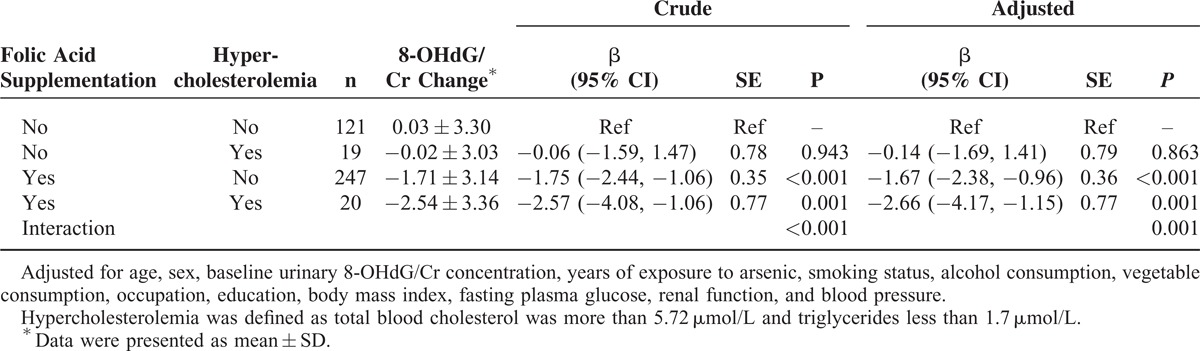
The Joint Effect of Folic Acid Supplementation and Blood Cholesterol Level on the Reduction of Urinary 8-Hydroxy-2’-Deoxyguanosine/Creatinine

## DISCUSSION

Our findings demonstrate that FA fortification is independently linked to the reduction of oxidative damage to DNA in a dose-related pattern and this effect is apparently stronger in those with hypercholesterolemia, which suggests that FA is beneficial to oxidative DNA damage reversal and jointly associated with blood cholesterol level.

Human first suggested in the 1950s that oxygen radicals induced cumulative damage.^16^ Since then, accumulating evidence has shown that ROS causes damage to DNA and other macromolecules, such as lipids and proteins.^16^ Reactive oxygen species can directly attack DNA, influence the DNA methylation and lead to oxidative DNA damage.^17^ Increased DNA damage triggers apoptosis and leads to various pathogenic states. It will largely influence numerous cellular processes linked to the development of aging and various major diseases such as diabetes, atherosclerosis, and cancers.^18^ Among more than 20 identified products of ROS-mediated DNA damage, a major one is 8-hydroxyguanine (8-oxoG) or 8-hydroxy-2’-deoxyguanosine (8-OHdG). 8-hydroxy-2’-deoxyguanosine is excreted through urine without being further metabolized, and its urinary appearance reflects oxidative DNA lesion and its repair.^3,19^ Because of its ease of detection, known potential mutagenic and abundance in DNA, 8-OHdG has been widely accepted as the specific biomarker of the “whole body” oxidative DNA damage and the cellular oxidative stress in the last several decades.^20^

Previous studies report that Hcy is independently linked to oxidative stress.^21,22^ Elevated Hcy is observed to be significantly associated with increased oxidative stress, which is one of the most important mechanisms contributing to Hcy-induced tissue injury.^23^ Furthermore, hyperhomocysteinemia (HHcy) may also induce increased oxidative stress,^24^ affect antioxidant defense systems^25^ and promoting DNA damage.^26^ As an antioxidant and key co-factor in one-carbon metabolism, folate is the major contributor of S-adenosylmethionine (SAM), which is the primary intracellular methyl donor.^27^ Meanwhile, DNA methylation also plays a key role in repairing oxidative stress mediated DNA lesions and is critically affected by SAM.^28^ Increasing evidences suggests that folate deficiency will lead to HHcy and largely influence DNA synthesis and repair.^21–23^ It may decrease DNA stability and increase the risk of malignant transformation, either by disrupting DNA methylation or perturbing the nucleotide pool, negatively altering DNA synthesis and repair, leading to altered gene transcription and proto-oncogene expression.^29^ Furthermore, independent of Hcy lowering effects, folate may also have protective effects through free-radical scavenging activity^30^ and endothelial dysfunction improving.^31^ Similar to the present study, Lee et al^14^ firstly reported that exogenous administration of FA is beneficial to reduce oxidative stress in ethanol-fed rats, an animal model of alcoholic liver disease.

Arsenic is a well-known potent environmental oxidative stressor in a number of countries.^11,32^ Inner Mongolia is one of the most serious water-caused arsenic exposure regions in China, with approximately 400,000 people obtaining their drinking water from private wells exposed to arsenic levels above 50 μg/L, and suffering various adverse health effects.^33^ Arsenic is metabolized in human beings mainly via methylation reactions, associated with 1-carbon metabolism and SAM as the methyl donor.^34^ Methylation of inorganic arsenic consumes a large amount of SAM, leads to HHcy, folate deficiency, and oxidative stress-induced DNA lesion. Long-term exposure to arsenic is independently associated with many major chronic diseases, such as diabetes and cancers.^34^ Our previous studies have also demonstrated that urinary 8-OHdG concentration positively correlates with chronic arsenic exposure.^35,36^ Oxidative DNA damage may be one of the most important mechanisms in the pathogenesis of arsenic-induced diseases.^35^ This is the main reason why we select the subjects in the present study from a population which has had long-term exposed to arsenic in Inner Mongolia.

It is well known that high cholesterol levels and oxidative stress all play important roles in the pathologies of atherosclerosis. Dimitrova-Shumkovska et al^37^ reports that a high fat a high cholesterol atherogenic diet leads to increased oxidative stress in the liver and aorta. Meanwhile, another study found that a high intake of saturated fatty acids is associated with high plasma concentrations of total Hcy.^38^ In the present study, where all of the subjects come from Inner Mongolia, China, too many high cholesterol foods are consumed in their daily life, and 9.51% of them have high blood cholesterol. We hypothesized that cholesterol metabolism might influence urinary 8-OHdG/Cr levels, as well as the effects of folate on cholesterol metabolism and modulating cholesterol levels on homeostatic responses to folate perturbation.^39^ Hence, we further analyzed the impact of blood cholesterol on 8-OHdG metabolism by comparing those subjects with or without high blood cholesterol levels in the study, and found a significant joint effect between FA and higher blood cholesterol for reduced urinary 8-OHdG/Cr levels. The findings suggest that cholesterol metabolism might have an impact on DNA oxidative damage prevention with FA. Participants with both DNA oxidative lesion and high blood cholesterol might need to be much more fortified with FA and to achieve greater efficacy of the lesion improvement. Our results provide new insights into oxidative stress mediated damage prevention and improvement.

The present study is the first, to the best of our knowledge, to examine the beneficial effects of FAS on oxidative DNA damage by assessing urinary 8-OHdG concentration and to ascertain whether there is a dose-response relationship between FAS and 8-OHdG level in urine. The main finding is that urinary 8-OHdG concentration declines significantly after FA intake during a period of 8 weeks after adjusting for the potential confounding effects, including the baseline urinary 8-OHdG/Cr concentration. In addition, a clear dose-response relationship was observed between the intake of FA and urinary 8-OHdG reduction. This confirms that folate is beneficial to protect against oxidative DNA damage. Finally, we also found a joint effect between hypercholesterolemia and oral FA administration on 8-OHdG/Cr reduction. Participants with hypercholesterolemia fortified with FA achieved greater efficacy than those with normal blood cholesterol.

### Strength and Weakness

The main strength of the present study is the highly robust effect of oral fortification with FA based on the randomized, double-blind, and placebo-controlled clinical trial. Our findings suggest that FAS could be incorporated into medical clinics for those with DNA oxidative damage, which can be identified by assessing their urinary 8-OHdG/Cr concentration, and be administered by trained staff in medical clinics. Another strength of our study is that we select urinary 8-OHdG as the surrogate endpoint of DNA oxidative lesion. Previous studies have shown that 8-OHdG is a widely accepted biomarker for DNA oxidative lesion, its appearance usually reflecting well the oxidative DNA damage in the whole organism, and is very stable during urine and assay, which can be detected easily even at low levels.

Because we could not find a widely accepted diagnostic criterion for DNA oxidative damage; we are not able to guarantee that all of the subjects in the present study have confirmed DNA oxidative damage. Some subjects without DNA oxidative lesion might decrease the efficacy of FAS on the repair of the damage. This may be a weakness in our hypothesis. Furthermore, only 20 subjects can be diagnosed as having hypercholesterolemia among participants receiving FAS. The low power induced by this small sample size may be the major reason why the difference in the change of urinary 8-OHdG/Cr between participants with normal or abnormal blood cholesterol does not achieve significant level (*P* = 0.175), although subjects with hypercholesterolemia have an added 0.96 ng/mg reduction in urinary 8-OHdG/Cr than their controls. This will certainly reduce the impact of blood cholesterol level on the efficacy of FA on oxidative DNA damage. Without these weaknesses, the efficacy of FAS on the repair of DNA oxidative damage will be improved and the impact of blood cholesterol on the repair will be more credible. Another possible weakness of this study may be that we use a commercial ELISA kit instead of HPLC to measure the urinary 8-OHdG concentration. High-performance liquid chromatography has been widely accepted as the gold-standard method in many laboratory tests in the last several decades. ELISA, however, is based on the specific interaction between antibody and the corresponding antigen, has significant advantages such as high sensitivity and specificity, simple sample preparation, high throughput, and low cost per sample, over traditional chromatographic methods, and is also widely applied as the main analytical method for the determination of both large and small analytes and even heavy metals in the biologic, medical, agricultural, and environmental fields.^40–46^ A recent study assessed the accuracy and precision of ELISA with HPLC in 7 spiked samples, which were simultaneously analyzed by ELISA and liquid chromatography coupled with tandem mass spectrometry (LC–MS/MS), and found that there was a high correlation coefficient of 0.9956 (n = 7) between the 2 methods.^47^ This study demonstrated that ELISA was a feasible quantitative/screening method.

In summary, the concentration of urinary 8-OHdG/Cr, the biomarker of DNA oxidative damage, is highly modified by oral FAS which indicates that FAS can be used to reverse oxidative stress-induced DNA lesions. This effect is jointly associated with high blood cholesterol levels. Further studies are needed to confirm our findings. We have not used the data from the serum folate and plasma Hcy in the present study because this kind of study has been reported extensively.

## CONCLUSIONS

The present study demonstrates that FAS is safe and beneficial for oxidative DNA damage reversal in a dose-response pattern. Participants with hypercholesterolemia show a stronger effect of FAS on oxidative DNA damage protection. We provide a new insight into the prevention and reversal of oxidative DNA damage.
